# Understanding the ZIF-L to ZIF-8 transformation from fundamentals to fully costed kilogram-scale production

**DOI:** 10.1038/s42004-021-00613-z

**Published:** 2022-02-09

**Authors:** Adam Deacon, Ludovic Briquet, Magdalena Malankowska, Felicity Massingberd-Mundy, Svemir Rudić, Timothy l. Hyde, Hamish Cavaye, Joaquín Coronas, Stephen Poulston, Timothy Johnson

**Affiliations:** 1 Johnson Matthey Technology Centre, Chilton Site, Belasis Avenue, Billingham, Cleveland, TS23 1LB UK; 2 Johnson Matthey Technology Centre, Blount’s Court, Sonning Common, Reading, RG4 9NH UK; 3 Instituto de Nanociencia y Materiales de Aragón (INMA), CSIC-Universidad de Zaragoza, 50018 Zaragoza, Spain; 4 Chemical and Environmental Engineering Department, Universidad de Zaragoza, 50018 Zaragoza, Spain; 5 ISIS Neutron and Muon Source, Rutherford Appleton Laboratory, Harwell Oxford, Didcot, OX11 0QX UK

**Keywords:** Metal-organic frameworks, Chemical engineering, Porous materials

## Abstract

The production of MOFs at large scale in a sustainable way is key if these materials are to be exploited for their promised widespread application. Much of the published literature has focused on demonstrations of preparation routes using difficult or expensive methodologies to scale. One such MOF is nano-zeolitic imidazolate framework-8 (ZIF-8) – a material of interest for a range of possible applications. Work presented here shows how the synthesis of ZIF-8 can be tracked by a range of methods including X-ray diffraction, thermo gravimetric analysis and inelastic neutron scattering – which offer the prospect of in-line monitoring of the synthesis reaction. Herein we disclose how the production of nano-ZIF-8 can be conducted at scale using the intermediate phase ZIF-L. By understanding the economics and demonstrating the production of 1 kg of nano-ZIF-8 at pilot scale we have shown how this once difficult to make material can be produced to specification in a scalable and cost-efficient fashion.

## Abstract (web summary)

The metal-organic framework ZIF-8 has demonstrated promise for a wide range of applications, but its synthesis typically involves methodologies that are difficult or expensive to scale up. Here the authors show how the production of nano-ZIF-8 can be conducted at the 1kg scale in an economical manner through the intermediate phase ZIF-L.

## Introduction

Metal-organic frameworks (MOFs) are an exceptional class of materials that have attracted much attention since their discovery 30 years ago. MOFs are formed by the self-assembly of metal ions or clusters, known as nodes, interconnected with organic molecules, known as linkers. Their synthetic versatility leads to an enormous amount of possible combinations of metal nodes and organic linkers, and results in many different structures. It is this versatility that makes them applicable to a number of different industrially relevant applications such as: gas separation^1–4^ and storage^5–7^, catalysis^8–11^, drug delivery^12–14^ and sensors^15–17^.

MOFs have attracted attention for gas capture applications; their high specific surface area, pore volume and narrow pore size in the range of microporous make them very good candidates in the separation of, for example, CO_2_ from other gases^4,18^. Typically for these applications MOFs are incorporated into polymers forming mixed matrix membranes^19^. In such a configuration, MOFs are used as a dispersed filler and a polymer works as a supportive matrix. The addition of MOFs increases gas permeance while retaining and, in some cases improving, separation selectivity. This happens mainly due to the MOF microporosity and good polymer-filler interaction that helps avoid non-selective gaps or cracks at the membrane structure, which is common for other type of fillers such as zeolites, graphene or silica. Moreover, it is possible to modify (by proper functionalization of a given MOF structure) the aperture size of the MOF, i.e., tuning it based on the gas mixture we would like to separate^20^. In order to achieve maximum separation efficiency, the membranes are formed into thin-film composite (TFC) layers where a non-selective supporting polymer is covered by a very thin (~300 nm selective membrane layer)^21^. MOF nano particles (NP) might be incorporated into this selective layer, as larger particles cause defects in the membrane surface reducing performance, leading to the formation of thin-film nanocomposite (TFN) membranes. Moreover, to improve the efficiency of performance, membranes could be formed into a hollow fibre configuration with MOF incorporated on the selective skin of the membrane^19,21,22^.

**Fig. 1:**
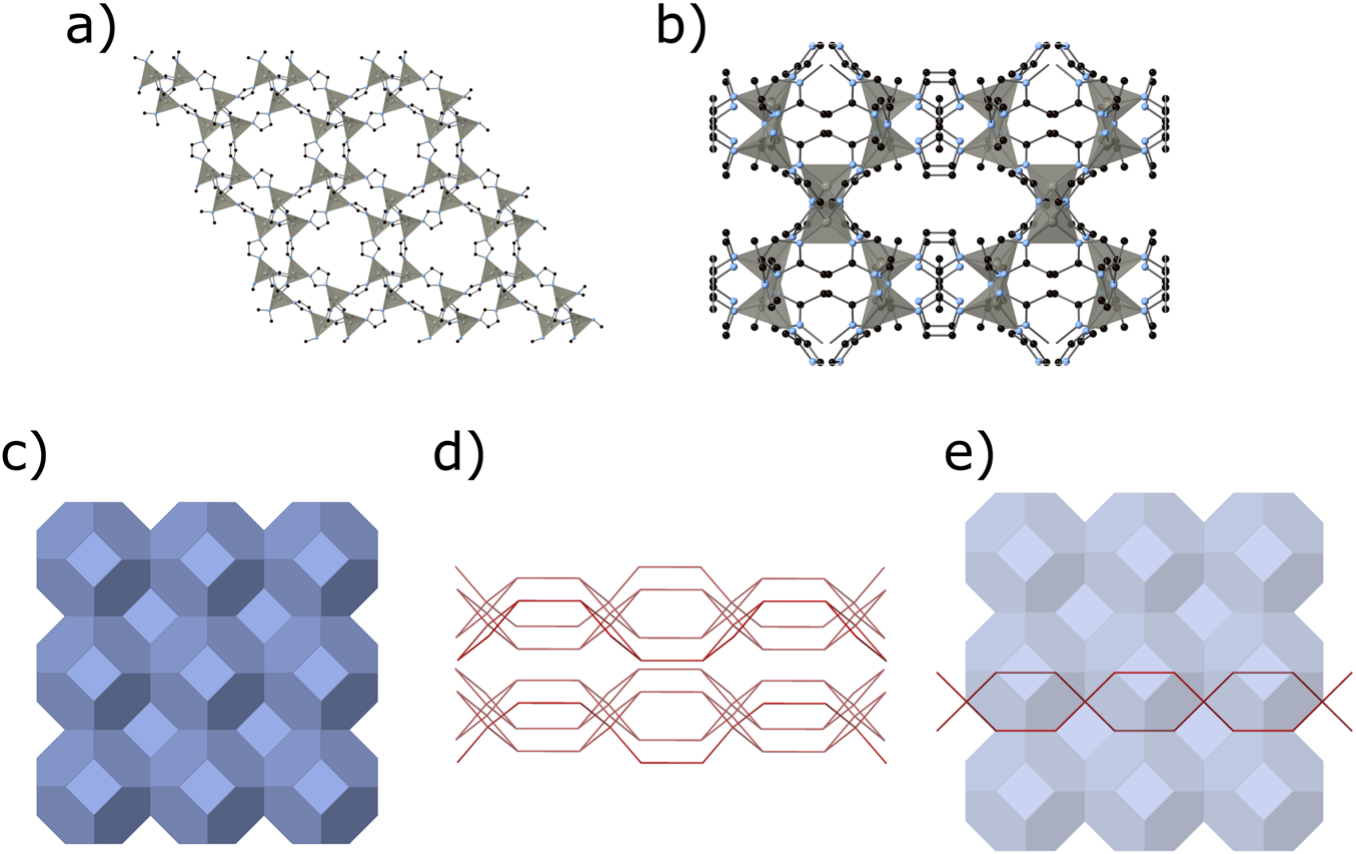
Graphical representations of the ZIF-8 and ZIF-L crystal structures. The structures of **a** ZIF-8 and **b** ZIF-L. Black spheres represent carbon, blue represent nitrogen and grey tetrahedra represent ZnN_4_. Hydrogen atoms omitted for clarity and the underlying topological units for **c** ZIF-8, **d** ZIF-L and **e** the two superimposed at scale. Produced using Crystal maker X 10.4.6 using CIF files from refs. ^35,43^.

Zeolitic imidazolate frameworks (ZIFs) are a sub-class of MOFs that are morphologically similar to zeolites. ZIFs are comprised of tetrahedrally coordinated metal ions linked by imidazole derivatives and they can be synthesised by various methods: solvothermal^23,24^, hydrothermal^25–27^, sonochemistry, precipitation^24,28,29^, mechanochemical^24,30^ or flow chemistry^31,32^. ZIF-8 (Fig. 1a, c), which is a well-known type of ZIF in the form [Zn(mim)_2_] (mim = 2-methylimidazolate), is comprised of Zn atoms linked via mim linking units forming a sodalite topology (SOD) with a pore aperture of 3.4 Å^33^. On the other hand, another type of ZIF: ZIF-L (Zn(mim)_2_·(Hmim)_1/2_·(H_2_O)_3/2_ or C_10_H_16_N_5_O_3_/2Zn) (Hmim = 2-methylimidazole) is closely related to ZIF-8 consisting of the same Zn and mim building blocks. ZIF-L produces leaf-shaped particles and has previously been investigated for CO_2_/CH_4_ separation^34^. Contrary to ZIF-8, ZIF-L (Fig. 1b, d) is formed from two-dimensional layers consisting of two, crystallographically different Zn ions, fully bridged mim, monodente Hmim and free Hmim molecules between the layers. This results in a partial SOD type topology with a cushion-shaped cavity of 9.4 x 7.0 x 5.3 Å^34^. The structure is stabilised by the intertwining layers of the monodente Hmim and free Hmim molecule through hydrogen bonding^34^. Fig. 1e shows the underling topological relationship between ZIF-L and ZIF-8. Crystallographic data for both phases can be found in Table 1.

**Table 1:** Crystallographic information for ZIF-8 and ZIF-L.

Name	Space Group	*a* (Å)	*b* (Å)	*c* (Å)	Density g/cm^3^	ref.
ZIF-8	*I*-43m	16.8815 (5)	16.8815 (5)	16.8815 (5)	0.9426	^67^
ZIF-L	*Cmca*	24.11910 (46)	17.06045 (33)	19.73984 (37)	1.4042	^34^

While a significant body of work has been created, much has focused on small scale proof of concepts, thus, further work is needed to develop MOFs for industrial uses. Large scale, green, economic routes need to be developed. Simply scaling-up synthesis routes developed in academic laboratories is not always possible. These routes often use toxic and expensive solvents, have under explored critical reaction parameters needed for scaleup and lack morphology control. Replacing toxic solvents with greener alternatives is required in order to make the routes more amenable to large-scale production and to develop a more sustainable process. Several articles have demonstrated greening of MOF syntheses either by replacing toxic solvents with more benign alternatives or increasing the reaction concentration, thus improving the space-time yield^35–37^.

ZIFs have been shown to undergo phase transformation between different morphologies as it is seen in the case of the thermal transformation of ZIF-7^38^ or the transformation of ZIF-L-Co to ZIF-67^39^ by a solvent mediated route. Moreover, ZIFs can undergo reversible phase transformation with high pressure known as the breathing effect as seen in the case of a number of ZIFs^40–43^. The transformation of ZIF-L to ZIF-8 was first reported by Low et al.^44^. The Authors direct the reader to Low et al. for a full explanation of the proposed mechanism and further discussion of the structure of the two frameworks. In brief, the mechanism is described as a topotactic transformation occurring via a geometric contraction model. Solvent molecules interact with Hmim molecules between layers, breaking and reforming hydrogen bonds, displacing the layers away from each other. Two transformation pathways were proposed for the transformation, sliding along the (001) plane in the [100] direction representing an overall displacement of 12.37 Å or sliding of two adjacent layers in the [010] direction with an overall displacement of 6.95 Å.

Reported syntheses of nano ZIF-8 require dilute conditions resulting in vast excess of methanol^28^ being used. This is only compounded when trying to scale up this reaction. Methanol is classed as problematic in some solvent choice guides, scoring negatively in both environmental and health categories^45^. Synthesising nano ZIF-8 via the transformation of ZIF-L is a desirable method to produce nano ZIF-8 as much lower volumes of water and 2-propanol can be used improving both environmental and health impacts^45^.

The objective of this work is to explore the transformation of ZIF-L to ZIF-8 using a range of techniques. This fundamental understanding will then be used to demonstrate a scalable route to synthesise nano ZIF-8 from the transformation of ZIF-L and ultimately produce 1 kg of nano ZIF-8 using this route. The route reported here is economic, scalable and uses industrially available, greener solvents. Finally, the synthesis route developed has a potential to be used industrially and we aim to show how nano ZIF-8 could be produced industrially at 15 tonnes per year.

## Results and discussion

**Fig. 2:**
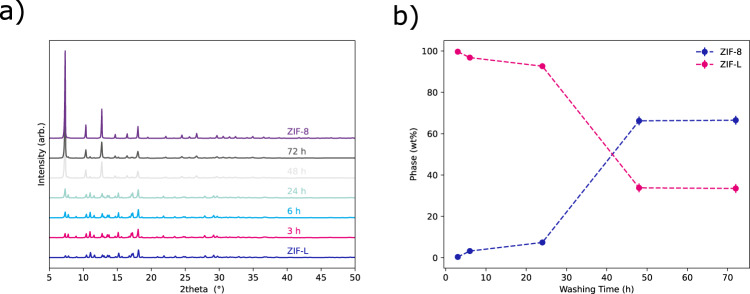
Diffraction data showing the phase change. **a** PXRD patterns of as made ZIF-L and samples washed at 3–72 h, respectively. Also included is a reference pattern of ZIF-8 made by literature routes. **b** Phase percentages calculated from Rietveld refinements of ZIF-8 and ZIF-L for samples washed.

The successful transformation of ZIF-L to ZIF-8 can be observed using PXRD (Fig. 2a). Peaks indexable to ZIF-L can be seen to diminish as diffraction of ZIF-8 is seen to become more prevalent during the 72 h experiment. Diffraction from ZIF-L is still observed in the material washed after 72 h. This can be seen in Fig. 2b where, after Rietveld Refinements (Supplementary Fig. 1), weight percentages of each phase were calculated. This shows that there is an induction period, up to 24 h, before a rapid phase transformation is observed. After 48 h no further transformation is seen, suggesting a ZIF-L core remains surrounded by ZIF-8 which would hinder the proper diffusion of reagents to/from the untransformed ZIF-L. The low intensity of Bragg diffraction observed for samples washed with methanol at 48 to 72 h seems, initially, at odds with the phase weight percentages obtained from the refinements. It should be noted that ZIF-L (1.4 g cm^−3^) is denser than ZIF-8 (0.94 g cm^−3^) resulting in even low-intensity reflections accounting for a large wt% of the sample.

**Fig. 3:**
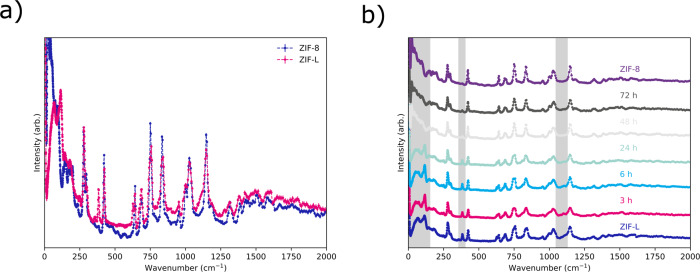
INS data showing the phase change. **a** INS spectra collected on reference ZIF-8 and as synthesised ZIF-L. **b** INS spectra of samples of ZIF-L after various washing times with methanol. Grey areas are for visual guidance only. Also included is a reference pattern of ZIF-8 made by literature routes.

Samples of reference ZIF-8, as made ZIF-L, and washed ZIF-L samples were subjected to inelastic neutron scattering (INS) experiments. Reference ZIF-L and ZIF-8 spectra can be seen in Fig. 3a. It is clear from these data that difference between ZIF-L and ZIF-8 can be observed in the collected INS. This can be seen in Fig. 3b where character from ZIF-L can be seen to diminish as ZIF-8 material is produced. To determine what modes these observed vibrations correspond to the vibration spectra of both materials were computed using density functional theory (DFT). The computed vibration spectra are in good agreement with the respective experimental vibration spectra from INS (Supplementary Fig. 2), indicating that our DFT parameters are converged enough to correctly reproduce and attribute the vibration modes of the structures.

**Fig. 4:**
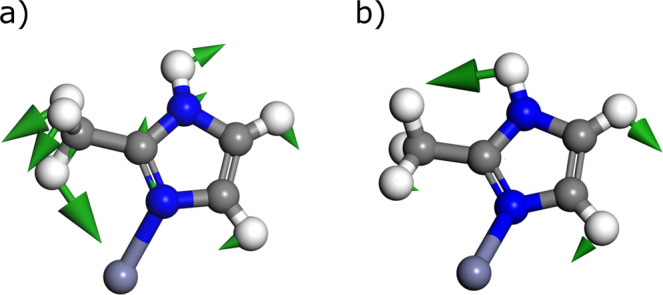
Graphical representation of highlighted vibrational modes. Vibration modes at **a** 365 and **b** 1063 cm^-1^ as computed by DFT. For clarity only the singly bound 2-methylimidazolate linker that are vibrating are shown. Grey spheres represent carbon, blue represent nitrogen, white represent hydrogen and lilac represent zinc. Green arrows indicate the direction and intensity of the vibration motion for each atom.

Figure 3b shows vibrational modes highlighted by the grey areas that can be attributed using DFT. While the vibrations modes below 200 cm^−1^ are difficult to confidently attribute using DFT due to the shallow energy potential surface, the vibrations modes at higher frequencies such as the ones computed at 365 and 1063 cm^−1^ are clearly attributed to singly bound 2-methylimidazolate linkers in the ZIF-L materials—Fig. 4a, b, respectively. These data are consistent with the published solved crystal structure and indicate that spectroscopy, not just diffraction, can be used to track this transformation. Supplementary Fig. 3 shows how the ratio of intensity of peaks at 280 cm^−1^ and 380 cm^−1^ can be used to track the transformation. A similar delay period is observed when compared to the Powder X-ray diffraction (PXRD) data, Fig. 2b. As ZIF-L is also related to the SOD topology with the 2D layers bridged by N-H···N hydrogen bonds of non-coordinating Hmim species^46,47^, the just mentioned change in intensities may deal with the disappearance of such hydrogen bonds when ZIF-L is converted into ZIF-8, where only deprotonated ligand is coordinated to Zn cations.

While it may not be practical to use neutron spectroscopy to determine phase percentages, it does indicate that IR or Raman experiments could be used for this in the future without the need of specialist XRD fitting software. This offers the tantalising possibility of in-line spectroscopy being used to track the transformation of ZIF-8 from ZIF-L during large-scale production of the framework. This is due to the INS spectra containing contributions for both IR and Raman active bands.

It should also be noted that a size difference is observable between the samples of ZIF-8 made in this study and those produced in previous literature reports^28^. This is likely due to the 10 x increase in scale. With such changes reaction variables like stirring and separation may lead to significantly different products. While a study on the effect of scale on these materials is a key, and currently underexplored, area of study it is outside the scope of this work.

**Fig. 5:**
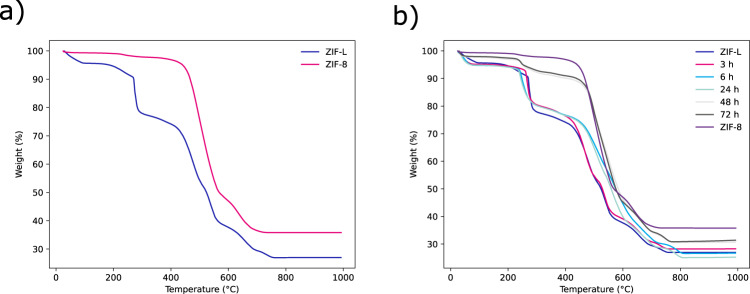
TGA data collected on samples obtained during the transformation. TGA (in air) traces for **a** ZIF-L and ZIF-8 and **b** samples subjected to washing.

To further understand this transformation, the samples previously analysed by INS and XRD were subjected to thermal gravimetric analysis (TGA). Figure 5a shows TGA weight loss curves for ZIF-8 and ZIF-L. When ZIF-8 is heated under flowing air between room temperature and at ~300 °C a drop of 2.3% is observed. As the onset of the weight loss is above 100 °C we suggest that this loss is due to residual linker still present rather than solvent loss. The largest weight loss is seen above 400 °C. This can be attributed to the destruction of the framework and a final residual weight of 35.9% can be observed. This is in good agreement with theoretical values of 35.8%—assuming the formation of ZnO from the empirical formula of the MOF mentioned above.

The TGA trace for ZIF-L (Fig. 5a) shows significant differences to the TGA trace for ZIF-8. Initially a 4.3% loss due to trapped solvent is visible. A second step is observed at 180 °C, which is in-line with the loss of linker (i.e., interlayer Hmim, not structural imidazolate) observed in the ZIF-8 trace. This loss attributed to the linker is significantly larger than that of ZIF-8 – with a loss of 18.5%. This is followed by framework degradation above 400 °C to a final residual weight of 26.9%. This is in good agreement with theoretical value of 27.5% from the empirical formula of ZIF-L seen above.

Figure 5b shows how, with increased washing time, the TGA traces collected develop stronger ZIF-8 character and weaker ZIF-L character. Importantly a reduction in weight loss is associated with the free linker within the gallery spaces of ZIF-L. Thus, the lower the ca. 180 °C step (related to the protonated ligand), the more progressed the transformation of ZIF-L to ZIF-8 is.

**Fig. 6:**
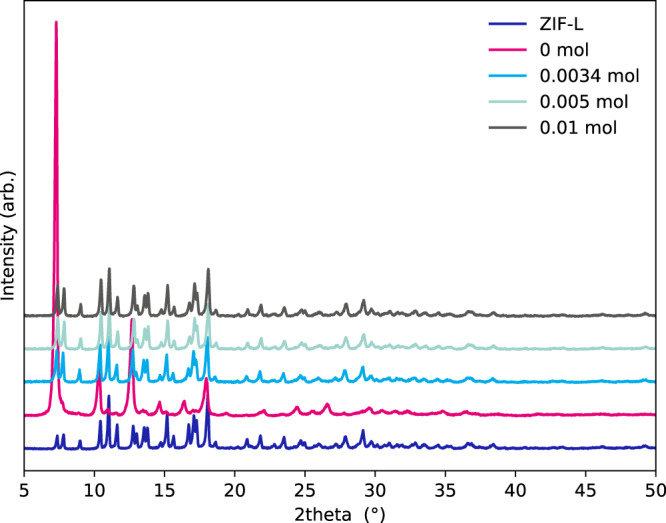
Diffraction data from samples washed with additional linker. PXRD patterns of samples washed for 24 h with various quantities of 2-methlyimidazole added.

To elucidate further on the mechanism of transformation, a series of experiments were conducted on a separate batch of ZIF-L from previous data shown. In these experiments free imidazole was added to the solvent used in the transformation. Figure 6 shows the PXRD patterns obtained from these samples with a total wash time of 24 h. The addition of excess linker hinders the transformation with any additional linker halting the transformation of the framework. We propose this is due to an unfavourable concentration gradient of the linker in the wash solvent. This would hinder the removal of the linker from the pore, hence inhibiting the transformation.

These washing experiments were conducted on newly prepared samples. It can be seen how after 24 h of washing a significant amount of ZIF-8 is produced, unlike previous samples, which required in excess of 24 h to see the transformation. This indicates that the induction period observed may be dependent on the sample itself and how much linker is present in the solvent used for transformation. As the transformation is linked to the removal of the one linker molecule for every two Zn atoms from the galleries of layered ZIF-L, we propose this is due to the ability of the solvent to remove the linker associated to the dissolution of at least part of the ZIF-L to favour its transformation into ZIF-8. This is in agreement with the topotactic transformation previously proposed by Low et al.^44^.

**Fig. 7:**
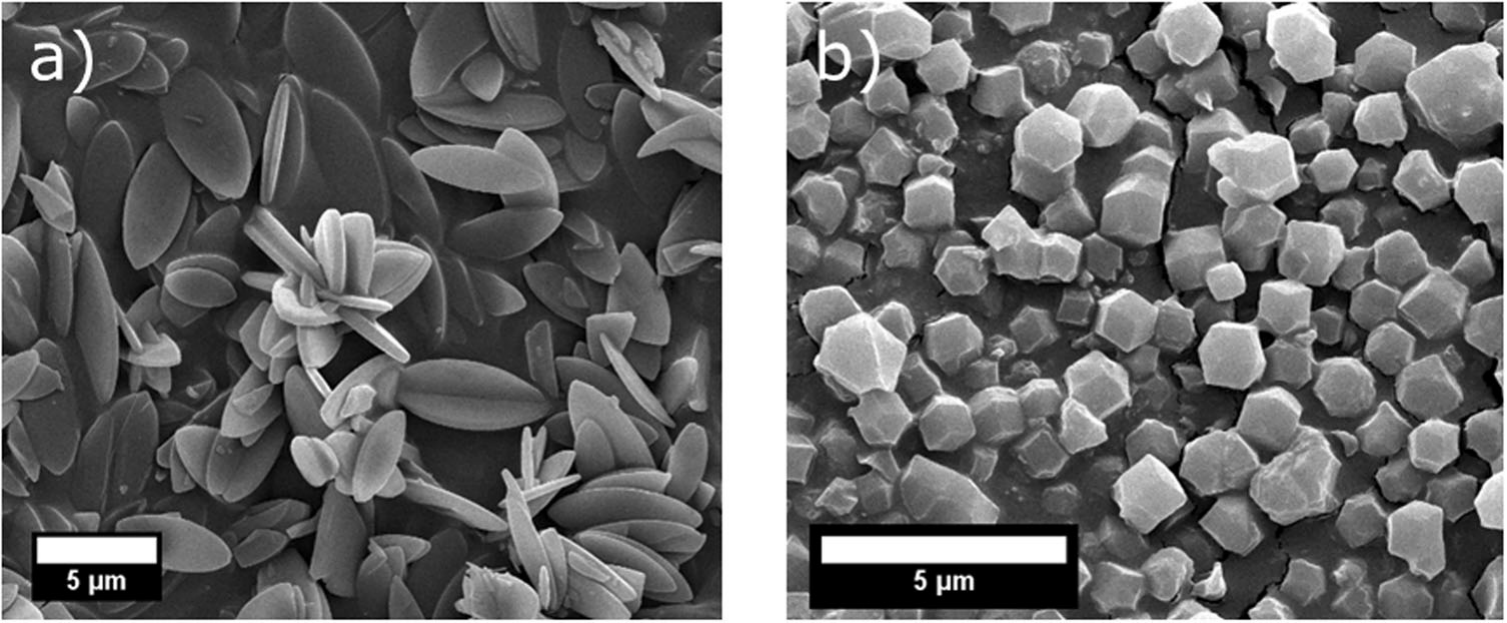
SEM images of ZIF materials. SEM micrographs of **a** as made ZIF-L and **b** ZIF-8.

However, typical topotactic transformations (i.e., structural transition, including or not material loss or gain, between two crystalline phases with one or more crystallographic equivalences), like those of layered zeolites^48^ may not produce such a large change in crystal habit growth. In fact, further evidence of this dissolution—recyclization route can be seen in SEM images. Figure 7a shows the characteristic leaf like morphology of ZIF-L. This, as well as the range of particle size (ZIF-L are ca. 5 µm in size, while ZIF-8 ca. 1 µm), can be contrasted against the orthorhombic particles observed for as made ZIF-8, Fig. 7b. In any event, the dimension of the ZIF-8 particles is approximately in the order of that corresponding to the thickness of the ZIF-L leaves.

SEM images of samples taken during the washing progresses from 6 to 72 h show leaf-shaped particles disintegrate (ZIF-L agglomerates disappear) and small particles can be observed on the surface of the once pristine crystals (Supplementary Fig. 4). After the previously discussed induction period the particles can be seen to no longer contain leaf-shaped particles and poorly defined particles of ZIF-8 have replaced the leaf particles of ZIF-L. It is curious that no intermediate/mixed morphologies between both phases are shown in these SEM images. This may be visible in intermediate sample between 24 h and 48 h. Nevertheless, 3D (ZIF-67, a ZIF-8 isostructural with Co instead of Zn) and 2D (Co-ZIF-L) morphologies were simultaneously obtained under certain crystallisation conditions depending on the methanol to water ratio used, ZIF-L being obtained only when the solvent was water or if the methanol content was low enough^47^.

**Fig. 8:**
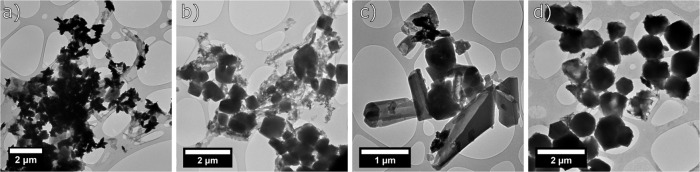
TEM Images of washed materials. TEM images of samples washed at **a** 6 h, **b** 24 h, **c** 48 h, and **d** 72 h.

TEM images of the selected samples, i.e., washed at 6 h (Fig. 8a), 24 h (Fig. 8b), 48 h (Fig. 8c) and 72 h (Fig. 8d) confirm the presence of ZIF-L and ZIF-8 and the transition from one ZIF to another. After 24 h of washing, the TEM image shows that the nanoparticles start to be randomly oriented and that gradually higher number of smaller NPs appear that can correspond to ZIF-8. It is also visible how large platelets are transforming into smaller NPs.

Moreover, two additional methods were used to probe the presence of the two MOFs: selected area electron diffraction (SAED) and Fourier transform. Both methods are complementary in measuring and calculating the d-spacing from Bragg’s law, Equation (1).

1 $$2sin\theta = n\lambda$$

Where, n is a positive integer, *λ* is the wavelength of the incident wave, θ is the angle and d the separation of atomic layers.

**Fig. 9:**
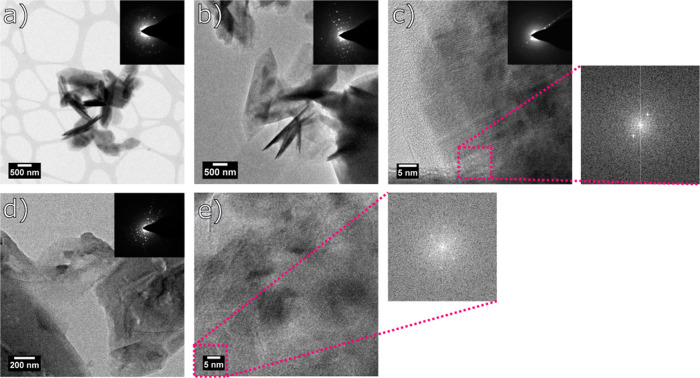
TEM micrographs and electron diffractograms of the washed samples. TEM images of samples at different **a** no washing ZIF-L sample, **b** 6 h washing, **c** 24 h washing, **d** 48 h washing and **e** 72 h washing times with their corresponding SAED and/or Fourier transform measurements.

As shown in Fig. 9a, b, c and d (insets), clear diffraction spots confirm the crystalline structure of the MOF nanoparticles. The average d-spacing calculated from Fig. 9a is equal to 4 Å, which corresponds to (114) crystal lattice plane using Miller (*hkl*) indexes that indicates the presence of ZIF-L. It is observed that with an increase of the washing time, the content of ZIF-8 starts to increase. Thus, after 6 h of washing the composition of the sample is a mixture of ZIF-L and ZIF-8. This is confirmed by the calculation of d-spacing average values equal to 3 Å that correspond to (443) lattice planes (ZIF-8), 8.5 Å and (002) lattice planes (ZIF-L) and 5.3 Å corresponding to (013) for ZIF-L and (023) for ZIF-8. Figure 9c, e show the d-spacing measurement by SAED and Fourier Transform. The pink box indicates the region chosen for the calculation of the transform. Some of the samples present either only SAED analysis or Fourier analysis, the reason for that is the difficulty in measurement due to samples instability under the beam. The last sample of 72 h of washing presents the d-spacing of 8.7 Å corresponding to (020) crystal lattice (ZIF-8) and a mixture of both MOFs with d-spacing of 4 Å (crystal lattice (114) for ZIF-L and (314) for ZIF-8)^44^.

### Scaleup

As stated above the production of nano-ZIF-8 is hindered by large volumes of organic solvent. This is a concern for both the safety and environmental impact but also the cost of production. To determine if the ZIF-L to ZIF-8 route explored above could be used in the production of nano-ZIF-8 a large pilot reaction was conducted. Here nano-ZIF-8 was produced via the ZIF-L route at a 10 L scale. This route ultimately produced 1 kg of nano-MOF—a yield of 81% based on zinc.

**Fig. 10:**
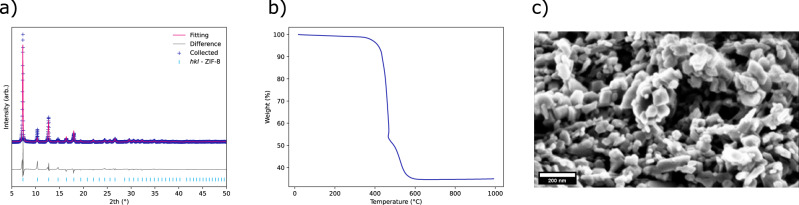
Various data collected on the large-scale batch. **a** Collected PXRD pattern and subsequent Rietveld refinement, **b** TGA trace of nano ZIF-8 material and **c** SEM micrograph taken of nano ZIF-8 material all from the one-kilogram batch of nano ZIF-8 produced via the transformation from ZIF-L.

PXRD and subsequent refinement of collected data for this sample can be seen in Fig. 10a, which shows a phase pure material is obtained from this large-scale reaction.

Figure 10b shows how the TGA trace for the large-scale sample is consistent with ZIF-8. A large thermal decomposition indicates full framework destruction occurs at ~400 °C. A final residual weight of 35.4% was recorded. This is in good agreement with theoretical values of 35.8%—assuming the formation of ZnO.

SEM micrograph, Fig. 10c, shows that nano morphology (average particle size ca. 65 nm) is obtained from this reaction. As discussed above, differences in scale can cause significant changes to the end product as seen when comparing Fig. 10c with the small scale ZIF-8 seen in Fig. 7b. It is important to note that these differ from the well-defined crystals produced from the methanol route. Their production via the dissolution, recrystallisation mechanism does not offer heterogeneity in terms of the size and shape of the particles produced. To determine if these samples could be used in applications where primary particle size was a key metric, physisorption experiments were conducted. Table 2 shows how the collected BET specific surface areas for this route are well within specification. The corresponding isotherm plots can be found in Supplementary Fig. 5.

**Table 2:** BET surface area values for ZIF-L and ZIF-8 produced via the original and new route.

Material	BET surface area (m^2^g^−1^)
ZIF-8 (original route)	1651
ZIF-8 (1 Kg batch route)	1745
ZIF-L	93

### Industrial analysis

It has been demonstrated previously that various organic solvents can be used to conduct the transformation of ZIF-L to ZIF-8. While we have focused on ethanol up to this point changes were needed when increasing the scale from the bench to pilot. Ethanol has several major drawbacks when it comes to large-scale synthetic scale-up. One key reason why ethanol should be avoided is due to additional regulatory implications including the need to acquire licences to use pure ethanol bringing additional costs. Denatured ethanol may be used as a substitute however it is not known how the additives in denatured ethanol would affect the production method. Propanol can be used without regulatory restriction and is preferred at industrial scale.

**Fig. 11:**
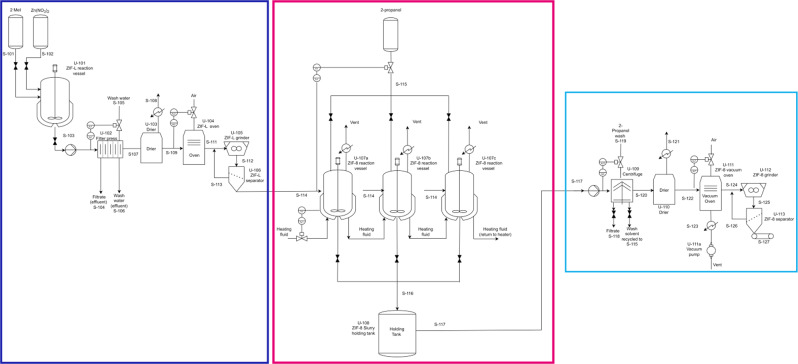
A proposed flow sheet. Proposed process flow concept diagram for nano ZIF-8 production. The dark blue box indicates the ZIF-L synthesis node, the pink box indicates the ZIF-L to ZIF-8 transformation node and the light blue box indicates the final nano ZIF-8 processing node.

Figure 11 shows a process flow concept for a nano ZIF-8 production plant using the ZIF-L transformation route. The process flow concept was designed using data from the scaled-up lab experiments. The concept was developed by separating out the different processes used in the lab synthesis and translating them to large-scale equipment. A materials balance from the start of the process to the end process was calculated and this was used to size components for the concept plant.

An estimate of component cost was made based on two methods, the first is a graphical method^49^ where a general relationship for cost versus size of component was determined by researching literature data. The second method is taken from Coulson and Richardson Volume 6^50^. This method again uses a general relationship for cost vs size of equipment. Chemical engineering plant cost index (CEPCI) values were used to convert historic costs to prices in 2019. The plant was sized to produce 15 tonnes of material per year, this is according to current estimated demand for use in mixed matrix membranes.

Fixed capital costs have been estimated by two methods and a final estimate will take an average of the two. The first estimate uses a method devised by Lang^51^, Eq. (2), where the fixed capital cost of the project is given as the total purchase equipment cost multiplied by a Lang factor.

2 $$Cf = {{{{{\mathrm{fL}}}}}}\cdot {{{{{\mathrm{Ce}}}}}}$$

Where Cf is the fixed capital cost, fL is the Lang factor and Ce is the total delivered cost of all major equipment items. The Lang factor chosen depends on the type of process (Supplementary Table 1), for the purpose of this estimation a Lang factor of 3.6 has been chosen to reflect a mixed fluids-solids processing plant.

The second factorial method (Supplementary Equation 1) considers direct-cost items (Supplementary Table 2) that are incurred in the construction of a plant, in addition to the cost of equipment. These additional costs and cost factors for materials of construction can be found in Supplementary Table 3. The contribution of each of these additional items to the total capital cost is calculated by multiplying the total purchased equipment by an appropriate factor. For the purpose of this estimation 316 steel has been chosen for piping and other components, a more detailed materials of interaction study should be completed for the next cost estimate. Using the current information, the capital expenditure for a plant of this is estimated to be around £1.3 million. Equations and values used to calculate fixed capital cost can be found in the supporting information.

Operating costs have been estimated based on a method found in Coulson and Richardson Volume 2^51^. Costs have been broken down into variable, fixed and other, a detailed breakdown and typical costs can be found in Supplementary Table 4. Annual production cost is calculated from the sum of the variable cost, fixed costs and other costs. Production cost per kilogram is calculated by dividing annual production cost by annual production rate in kilograms. Using this method an operating expenditure is estimated to be around £1.5 million per year and a production cost estimate of nano-ZIF-8 to be around £100 per kilogram.

**Fig. 12:**
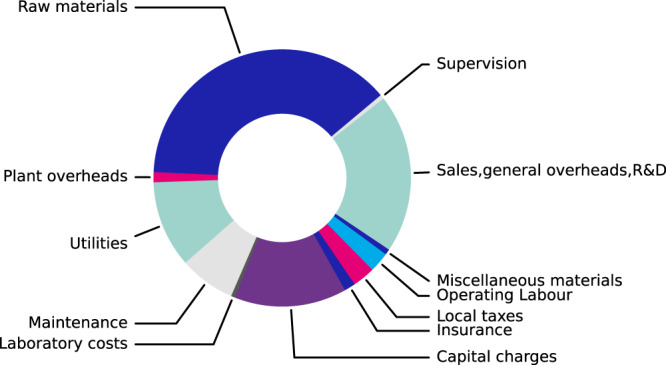
Cost estimate breakdown. Estimated cost distribution for nano ZIF-8 production.

Figure 12 shows a cost distribution estimate of the final material. The largest cost within the distribution corresponds to the raw materials used to produce nano-ZIF-8. This cost, however, could be reduced more by optimising the process further to include recycling of reagents^52^ and solvents used within the process, however as this was not studied during this work it was not included within this initial process flow concept. Although, raw materials make up the largest cost to produce the material it is not thought this is cost prohibitive to produce nano-ZIF-8.

This is a first assessment into scaling up this process, further work would be needed to develop detailed designs and more accurate estimates for the process. For instance, the use of alternative Zn salts^53,54^, following the same methodology developed here, could present some improvement from the point of view of both the design and sustainability of the process.

Comparing space-time-yields between the methanol route and the ZIF-L route shows significant improvements. An estimate of the scale-up production for the methanol route results in a space-time-yield of 4 kg m^−3^ day^−1^ while the ZIF-L route is able to produce nano ZIF-8 with a space-time-yield of 25 kg m^−3^ day^−1^ this is in-line with other MOF synthesis^55^.

## Conclusions

Nano ZIF-8 has been produced at the kg scale using a route utilising the transformation of ZIF-L to ZIF-8. This route offers considerable advantages over previous reports of the production of nano-ZIF-8 materials, notably requiring less solvent, an initial water-based reaction step and higher yields. Resulting in more material, made cheaper, with reduced environmental and safety concerns.

Herein we have disclosed how the industrially relevant transformation of ZIF-L to ZIF-8 can be tracked using a range of techniques that align well with previous reports. The new methodology produces a material with analogous textural and crystallinity properties than those achieved by the conventional route. In addition, we have demonstrated how INS can be collected and spectra predicted computationally, which show clearly how the transformation progresses via the reduction of singly bound imidazole in the pores. These data suggest the transformation could be tracked using in-situ spectroscopy techniques, which would be relevant in material scale-up.

The above data are in agreement with previous reports and confirm that the reaction mechanism occurs via topotactic transformation. The difference in induction period seen as well as data of washes with additional linker present indicate that this transformation is controlled by the concentration gradient of the linker within the wash solution.

More work is needed to understand if particle shape can be controlled using this route and also to further improve the green aspects of the procedure.

## Methods

### Materials

2-methylimidazole (Hmim) (99% purity), zinc nitrate hexahydrate (99 + % purity) and 2-propanol (99 + %) were purchased from Alfa Aesar, absolute ethanol (99.8 + % purity) was purchased from Fisher Scientific.

### Methods

#### ZIF-8 synthesis

ZIF-8 used in the study was produced as previously reported^28^. In brief zinc nitrate hexahydrate (29.33 g, 0.10 mol) was dissolved in MeOH (2 L). In a separate vessel, Hmim (64.89 g, 0.79 mol) was dissolved in methanol (2 L). The solution containing the linker was added to the metal and the resulting solution was stirred for 30 min. The precipitate was separated by centrifuge at 4000 rpm for 10 min and washed with methanol 3 times. The product was dried in ambient air for 16 h before activation under vacuum at 50 °C for 12 h.

#### ZIF-L synthesis

ZIF-L was produced using a previously reported method^34^. In brief, solutions of of zinc nitrate hexahydrate (26.6 g, 0.089 mol) was dissolved in water (190 mL) and Hmim (53.2 g, 0.64 mol) was dissolved in water as well (1.81 L) in a separate vessel. The linker solution was then added to the metal solution and then mixed under stirring at room temperature for 30 min. The product was collected by centrifuging at 4000 rpm for 10 min and the sediment was washed with water three times.

#### ZIF-L to ZIF-8 transformation

ZIF-L (2 g, 6.76 mmol) was dispersed in ethanol (200 mL) and heated to 60 °C for 72 h. This was left to cool before separation by centrifuge at 4000 rpm for 10 min. The sample was dried at 150 °C overnight under vacuum.

#### ZIF-8 Scaleup method

A kilogram batch of nano ZIF-8 was prepared using the following method:

ZIF-L was prepared by dissolving 2-methylimidazole (2216.7 g, 27 mol) in water (8 L) and zinc nitrate hexahydrate (1309.4 g, 4.4 mol) in water (1 L). The zinc solution was added to the imidazole solution and mixed at room temperature for 30 min using an overhead stirrer with an impeller mixing shaft. The product was collected by filtration and the solid residue washed three times with water.

The solid was dried in a vacuum oven at 60 °C overnight, once dried the solid was ground and sieved to < 500 µm. ZIF-L was transformed to ZIF-8 by dispersing in 2-propanol to make a 10 wt% solution. This suspension was heated to 80 °C for 48 h. Once complete the reaction was left to cool to room temperature before separation by centrifuge at 4000 rpm for 10 min. The resulting solid was washed 3 times with 2-propanol. The product was dried in ambient air for 16 h before activation under vacuum at 80 °C for 16 h.

#### Computational

INS spectroscopy was used to understand the atomic movements within a sample specifically to understand the changes observed as a phase change occurs, while ZIF-8 has previously been studied^56^ by INS, ZIF-L has not been reported. To this end computational simulations of both ZIF-8 and ZIF-L were conducted to allow for the identification of vibrational modes within a given sample.

All calculations were performed using the plane-wave Density Functional Theory (DFT) software CASTEP^57^ using the Perdew–Burke–Erzenhof (PBE) functional^58^. Core electrons were represented using the on-the-fly ultrasoft pseudopotential as available in with the Materials Studio package 2017^59^. A plane-wave energy cut-off of 570 eV was used for the valence electrons and the self-consistent field energy convergence threshold was set as 5.0 10^−7 ^eV/atom. As the unit cells considered are large, only the Gamma point was considered for the Brillouin zone sampling.

ZIF-8 was modelled using a 17.08 x 17.06 x 17.04 Å unit cell containing 240 atoms. The structure’s geometry was optimised using the Two-Point Steepest Descent (TPSD) algorithm^60^ until an energy threshold of 5.0 10^−6^ eV/atom, a force threshold of 0.01 eV/Å, an internal pressure threshold of 0.02 GPa and a displacement threshold of 5.0 10^−4^ Å were met.

ZIF-L was modelled using a large 24.51 x 17.20 x 19.88 Å unit cell containing 644 atoms. Due to the extensive number of degrees of freedom the geometry optimisation criteria were lowered to 10^−5^ eV/atom, 0.03 eV/Å, 0.05 GPa and 10^−3^ Å.

The phonon density of state of both structures was computed using the Finite Displacements method^61^. The interpolation was done using a q-vector grid spacing of 0.05 Å.

#### X-ray diffraction (XRD)

PXRD data were collected in reflection geometry using a Bruker AXS D8 diffractometer using Cu Kα radiation (*λ* = 1.5406 + 1.54439 Å) over the 5 < 2*θ* < 50° range in 0.02° steps, and a Bruker D2 Cu Kα radiation (*λ* = 1.5406 Å) over the 5 < 2*θ* < 50° range in 0.02° steps. Refinements were performed using Topas or GSAS-II^62^ with reflection profiles modelled using a fundamental parameters approach with reference data collected from NIST660 LaB_6_.

#### N_2_ adsorption

Samples were subjected to analysis by N_2_ physisorption measurements. Absorption and desorption isotherms were collected on a Micromeritics 3500 3Flex instrument. Experiments were conducted at 77 K for N_2_ isotherms. Isotherms were subject to BET analysis for surface area calculation.

#### Inelastic neutron scattering (INS)

INS was conducted on the TOSCA^63,64^ beamline TOF spectrometer at the ISIS Neutron and Muon source, Harwell, UK. The instrument resolution is ~1.25% of the energy transfer across the entire energy range. Each sample (ca. 5 g) was loaded into a pouch made from Al foil. This was sealed in a standard TOSCA flat aluminium cell. The sample was loaded into the spectrometer and sample environment was evacuated to 10^−5^ Pa. Each sample was measured for ~3 h until satisfactory statistics were achieved (ca. 500 µA). Data^65^ were collected and processed using MantidPlot 3.12.1^66^.

#### Scanning electron microscope (SEM)

SEM images of the nano-ZIF-8 and ZIF-L were obtained with FEI-Inspect F20 microscope operating at a voltage of 10 kV. All samples were coated with Au/Pd under vacuum conditions prior to analysis to inhibit sample charging and improve imaging of the samples.

#### Transmission electron microscopy (TEM)

TEM images were obtained with FEI Tecnai T20 transmission electron microscope operated at 200 kV. The drop of the sample dispersed in ethanol was placed onto a carbon mesh grid and left to dry before the observation under TEM.

#### Thermo gravimetric analysis (TGA)

TGA experiments were conducted on an TA Instruments SDT Q600 or Q650 TGA system. The samples were held in an alumina pan, the temperature was increased from room temperature to 800 °C or 1000 °C at a heating rate of 10 °C/min under a continuous air flow of 100 mL/min.

## Supplementary information

Supplementary Information

## Data Availability

Data collected on the TOSCA beamline is available at 10.5286/ISIS.E.RB1820437. Other data that support the findings of this study are available upon reasonable request from the corresponding author. Disclosure of these data are subject to permission from Johnson Matthey plc.
