# Supplementary material for: Understanding the ZIF-L to ZIF-8 transformation from fundamentals to fully costed kilogram-scale production
Source: Commun Chem. 2022 Feb 9;5:18. doi: 10.1038/s42004-021-00613-z (PMC9814364; doi:10.1038/s42004-021-00613-z)
Supplement: Supplementary file 1 — Supplementary Information [file 42004_2021_613_MOESM1_ESM.pdf]

Johnson Matthey
Inspiring science, enhancing life

Understanding the ZIF-L to ZIF-8 transformation from fundamentals to fully costed kilogram-scale production.

Authors: Adam Deacon[1], Ludovic Briquet[2], Magdalena Malankowska[3,4], Felicity Massingberd-Mundy[2], Svemir Rudić[5], Timothy I. Hyde[2], Hamish Cavaye[5], Joaquín Coronas[3,4], Stephen Poulston[2], Timothy Johnson[2,†].

Affiliations:
1 Johnson Matthey Technology Centre, Chilton Site, Belasis Avenue, Billingham, Cleveland, TS23 1LB, UK.
2 Johnson Matthey Technology Centre, Blount's Court, Sonning Common, Reading, RG4 9NH, UK.
3 Instituto de Nanociencia y Materiales de Aragón (INMA), CSIC-Universidad de Zaragoza, Zaragoza 50018, Spain.
4 Chemical and Environmental Engineering Department, Universidad de Zaragoza, Zaragoza 50018, Spain.
5 ISIS Neutron and Muon Source, Rutherford Appleton Laboratory, Harwell Oxford, Didcot, OX11 0QX, UK.

† Corresponding author: Timothy.Johnson@matthey.com

Contents

XRD refinements 2
INS 3
SEM Images 4
Adsorption Isotherms 4
Cost Analysis 5

XRD refinements

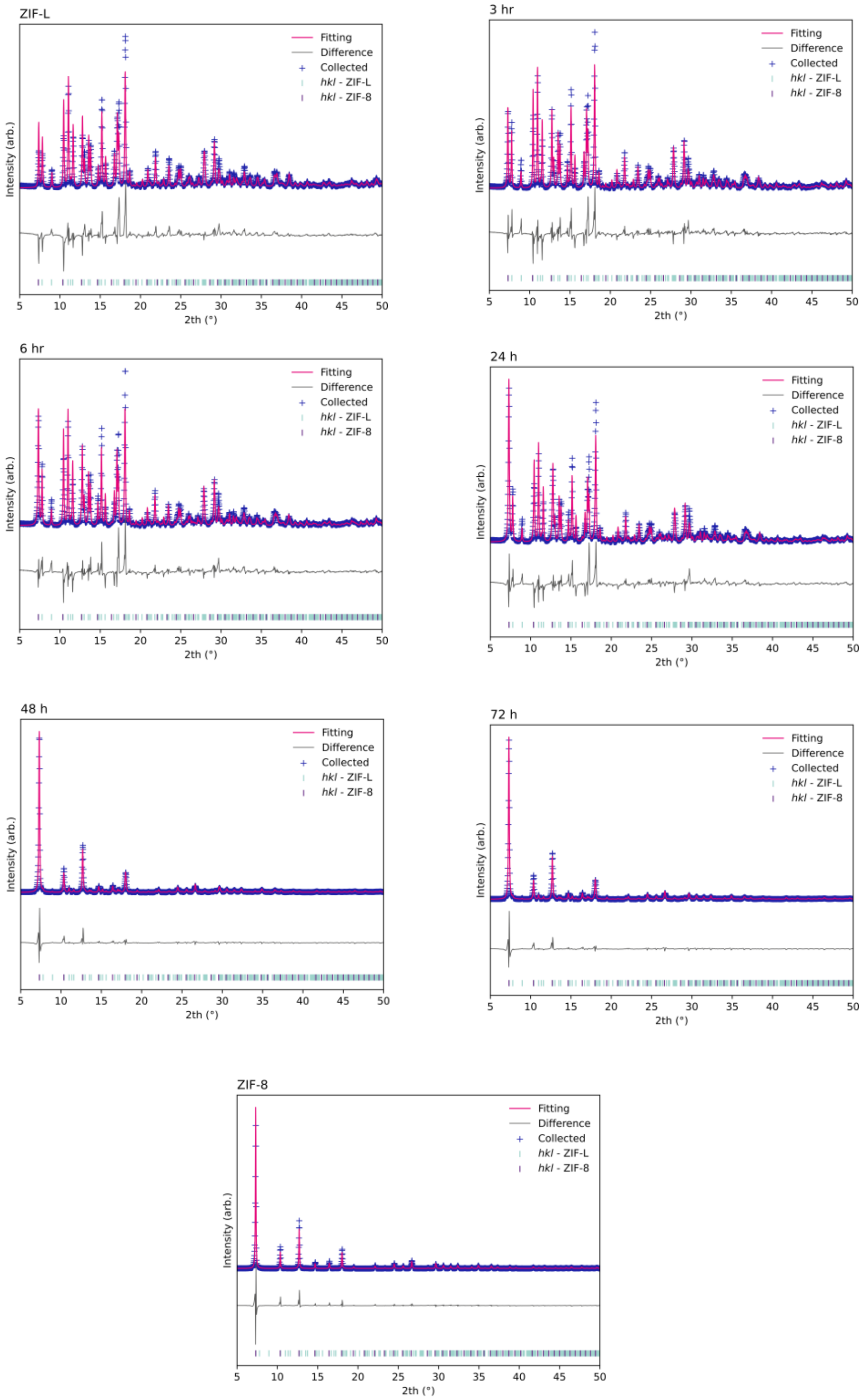

Supplementary Figure 1. PXRD patterns and subsequent Rietveld refinements for samples of as made ZIF-L, ZIF-L washed at various times and reference ZIF-8.

INS

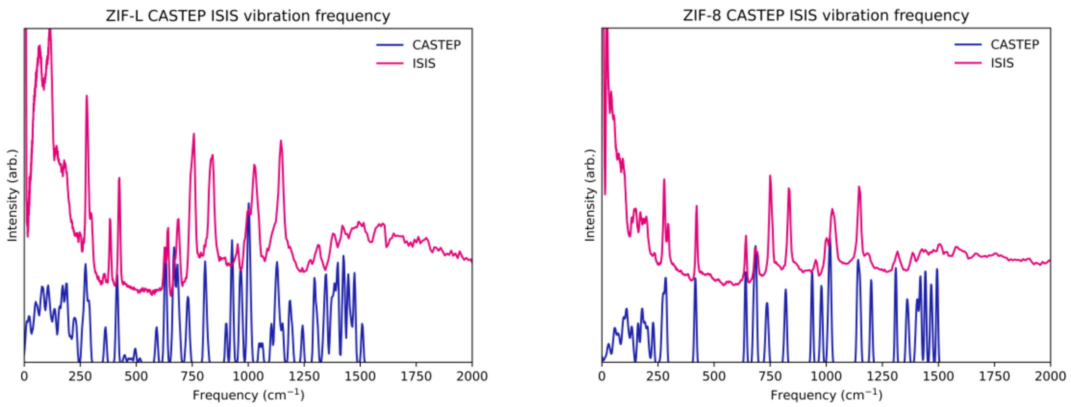

Supplementary Figure 2. Comparison of the ZIF-L (left) and ZIF-8 (right) vibration spectra as computed by DFT and as measured using INS.

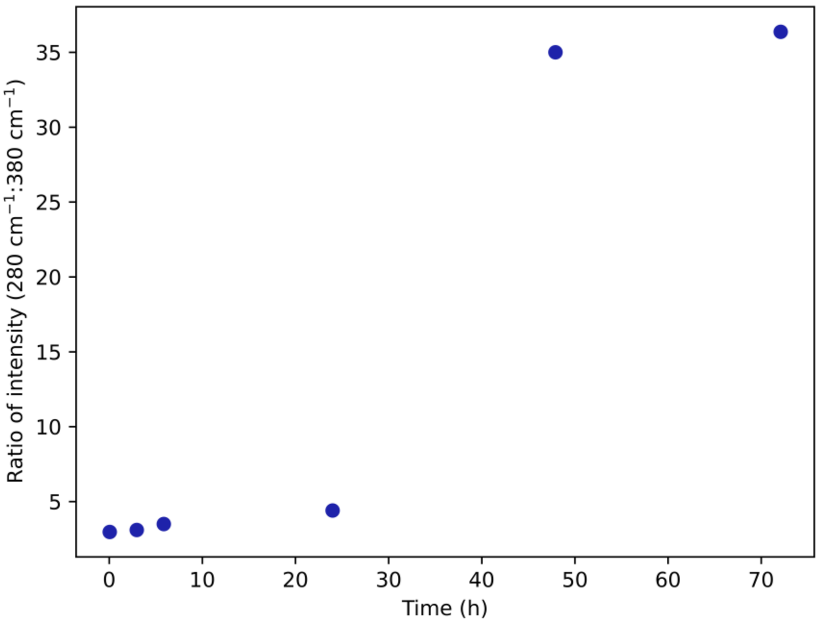

Supplementary Figure 3. Ratio of intensities of INS peaks at 280 cm-1 and 380 cm-1 as a function of time.

SEM Images

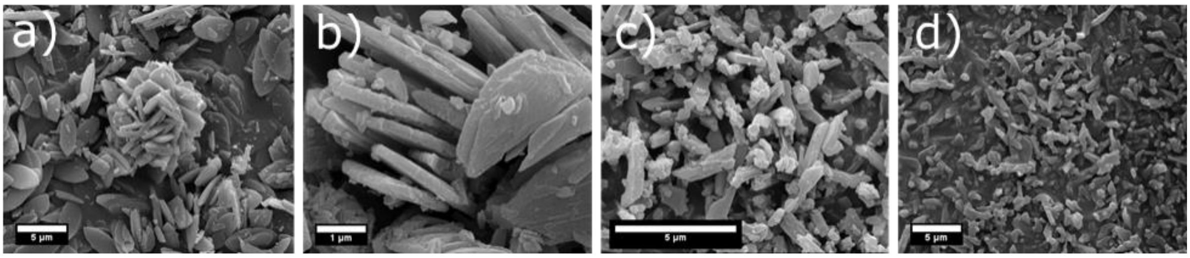

Supplementary Figure 4. SEM micrographs taken for samples washed at a) 6 h, b) 24 h, c) 48 h and d) 72 h.

Adsorption Isotherms

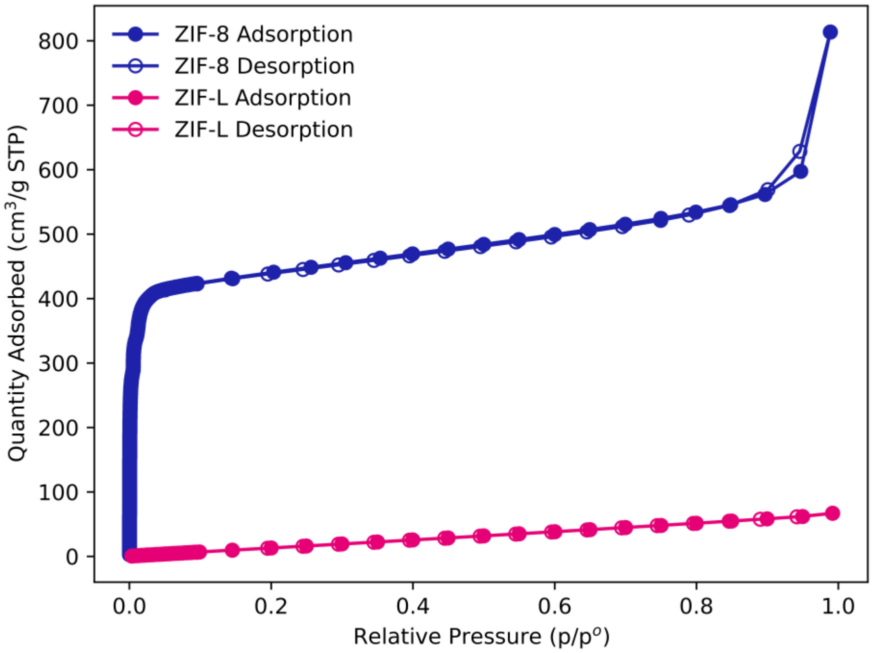

Supplementary Figure 5. BET isotherm plot of ZIF-L (bottom), and ZIF-8 material after transformation from ZIF-L (top).

Cost Analysis

Supplementary Table 1. Typical Lang factors for different plant processes.

Processing plant type	Lang Factor
Solids	3.1
Fluids	4.7
Mixed solids and fluids	3.6

Supplementary Equation 1.

$$Cf = Ce\,(1 + fp).fm + (fer + fel + fi + fc + fs + fl)$$

Supplementary Table 2. Direct cost items incurred in construction of a plant.

Factors for factorial fixed capital cost
fer - Equipment erection
fp - Piping
fi - Instrumental and control
fel - Electrical
fc - Civil
fs - Structures and building
fl -lagging and paint

Supplementary Table 3. Materials of construction cost factors.

**Variable costs**	**Typical cost**
Raw materials	From material balance
Miscellaneous materials	10% of Maintenance cost
Utilities	From material balance
**Fixed costs**	
Maintenance	5 % of fixed capital
Operating Labor	10 % of operating costs
Laboratory costs	20 % of operating labor
Supervision	20 % of operating labor
Plant overheads	50 % of operating labor
Capital charges	10 % of fixed capital
Insurance	2 % of fixed capital
Local taxes	1 % of fixed capital
**Other costs**	
Sales, general overheads, R&D	25 % of direct production costs

Supplementary Table 4. Production cost summary.

fm Materials factors

material	fm
Carbon Steel	1.00
Aluminium and bronze	1.07
Cast steel	1.10
304 stainless	1.30
316 stainless	1.30
321 stainless	1.50
Hastelloy C	1.55
Monel	1.65
Nickel and Inconel	1.70
